# Extreme Duty Cycles in the Acoustic Signals of Tiger Moths: Sexual and Natural Selection Operating in Parallel

**DOI:** 10.1093/iob/obaa046

**Published:** 2021-01-05

**Authors:** Y Fernández, N J Dowdy, W E Conner

**Affiliations:** 1 Department of Biology, Wake Forest University, 1834 Wake Forest Road, Winston-Salem, NC 27109, USA; 2 Department of Zoology, Milwaukee Public Museum, 800 West Wells Street, Milwaukee, WI 53233, USA

## Abstract

Sound production in tiger moths (Erebidae: Arctiinae) plays a role in natural selection. Some species use tymbal sounds as jamming signals avoiding bat predation. High duty cycle signals have the greatest efficacy in this regard. Tiger moth sounds can also be used for intraspecific communication. Little is known about the role of sound in the mating behavior of jamming species or the signal preferences underlying mate choice. We recorded sound production during the courtship of two high duty cycle arctiines, *Bertholdia trigona* and *Carales arizonensis*. We characterized variation in their acoustic signals, measured female preference for male signals that vary in duty cycle, and performed female choice experiments to determine the effect of male duty cycle on the acceptance of male mates. Although both species produced sound during courtship, the role of acoustic communication appears different between the species. *Bertholdia trigona* was acoustically active in all intraspecific interactions. Females preferred and ultimately mated with males that produced higher duty cycles. Muted males were never chosen. In *C. arizonensis* however, sound emissions were limited during courtship and in some successful matings no sound was detected. Muted and clicking males were equally successful in female mate-choice experiments, indicating that acoustic communication is not essential for mating in *C. arizonensis*. Our results suggest that in *B. trigona* natural and sexual selection may work in parallel, to favor higher duty cycle clicking.

## Resumen

La producción de sonido en árctidos (Erebidae: Arctiinae) juega un papel fundamental en la selección natural. Algunas especies de polillas utilizan los sonidos producidos por los órganos timbálicos como señales de interferencia para evitar ser depredados por los murciélagos. Llamadas con alto porcentaje de estimulación efectiva suelen ser más eficientes con este fin. Estos sonidos pueden ser utilizados también como forma de comunicación intraespecífica. Poco se sabe acerca de la función del sonido en la conducta de apareamiento de aquellas especies de polillas que utilizan estas señales, además, para interferir el sistema radar de los murciélagos. En este estudio, hemos grabado los sonidos producidos por dos especies de árctidos durante el cortejo, *Betholdia trigona* y *Carales arizonensis*. Ambas especies producen llamadas de alto porcentaje de estimulación efectiva como estrategia defensiva contra los murciélagos. Hemos caracterizado la variabilidad en las señales acústicas de ambas especies, se registró la preferencia de las hembras por las llamadas de los machos con diferentes porcentajes de estimulación efectiva y determinamos el efecto de las señales acústicas de los machos en la selección de pareja de las hembras. Aunque ambas especies produjeron sonidos durante el apareamiento, el papel de la comunicación acústica en este contexto difiere entre las especies. *Bertholdia trigona* produjo emisiones en todas las interacciones intraespecíficas. Las hembras de esta especie prefirieron y seleccionaron machos para aparearse capaces de producir señales con alto porcentaje de estimulación efectiva. Machos incapaces de producir sonido nunca fueron seleccionados para aparearse. En *C. arizonensis* por el contrario, las emisiones de sonido durante el apareamiento fueron escasas y en algunos casos no se detectó sonido. Las hembras no mostraron preferencia alguna por machos capaces de producir sonido, ni machos mudos, lo cual indica que la comunicación acústica no es esencial para el apareamiento exitoso de *C. arizonensis*. Nuestros resultados sugieren que en *B. trigona*, la selección natural y la selección sexual pueden actuar en paralelo para favorecer la producción de sonidos con alto porcentaje de estimulación efectiva.

## Introduction

Sexual selection molds male traits across a wide range of species (Greenfield 2016). Females respond to male variation by actively selecting males with preferred traits (Neelon et al. 2019). When the preference function is open-ended, sexual selection drives the evolution of selected traits toward more conspicuous and exaggerated signals until they become too costly to maintain. Thus, there is a trade-off, where sexual and natural selection functions antagonistically. Many studies have provided evidence of these two selective pressures working in opposition. African long-tailed widowbird males (*Euplectes progne*) possess a remarkable tail ornament under selection by females, where males with longer tails mate more successfully (Andersson 1994). However, flying with long tails is energetically costly and longer tails may make males more conspicuous in the open grassland and inhibit their flight performance during predator encounters (Pryke and Andersson 2002). Sexual selection and natural selection are at odds. Another example is the extinct Irish elk (*Megaloceros giganteus*) whose males sported enormous antlers that were once used during male–male competition for females. This sexually selected trait came at a cost; over evolutionary time males reallocated calcium from their bones to produce larger antlers (Emlen 2014). Again, sexual and natural selection acted antagonistically. In some moth species, including *Achroia grisella* (Pyralidae), the lesser wax moth, this antagonism is seen when acoustic signals produced by males both attract predators (gleaning bats) and mates (Rodriguez and Greenfield 2004).

**Fig. 1:**
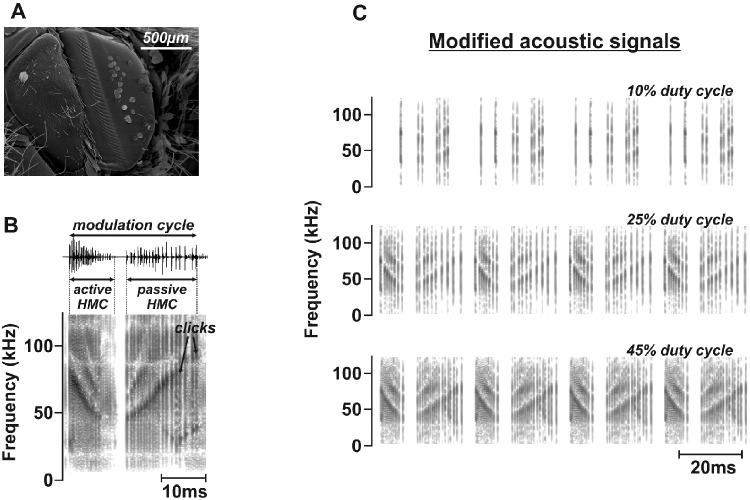
Tymbal morphology and acoustic emissions of *B. trigona*. (**A**) Scanning electron micrograph of the metathoracic tymbal organ. Image is oriented with the dorsal side up and anterior side toward the left. (**B**) Oscillogram and spectrogram of a single MC of *B. trigona*. Broadband clicks are produced by the sequential buckling of the tymbal organ (striated band in A) inward and outward to create the active and passive half-MCs (HMC). (**C**) Spectrograms of the artificially generated acoustic signals used as stimuli during female preference experiments. Signals were modified to generate 10%, 25%, and 45% duty cycles.

We here explore the consequences of a system involving tiger moths in which, unlike the above, sexual and natural selection may work in concert, not antagonistically. In the tiger moths *Bertholdia trigona* and *Carales arizonensis*, tymbal sounds jam bat echolocation causing bats to miss their target—natural selection—and herein we explore the role of tymbal sound in female mate choice—sexual selection. The sounds produced by arctiines consist of a train of broadband clicks produced during the flexion and relaxation of metathoracic tymbals with a silent interval between Fig. 1A and B. The moth ear is not capable of encoding the temporal detail (individual clicks) of the tymbal sound (Sanderford et al. 1998). Further, the sounds produced during defense and sex are identical. For bats the clicks are resolvable and important. The effectiveness of sonar jamming requires moth clicks to arrive at the bat’s ear within a short time window just prior to the arrival of an echo (Miller 1991; Tougaard 1998). Arctiine signals produced at high duty cycles (i.e., percentage of time occupied by sound in a 100 ms time window) would have a higher probability of falling inside the critical jamming window and effectively interfere with bat sonar. This relationship has been verified in free flying bats where duty cycle is correlated with making bats miss their targets (Corcoran and Conner 2012; Y. Fernandez et al. this issue). These findings suggest that bat selective pressure has driven the evolution of duty cycle toward higher values in sonar moth jamming species. It is conceivable that females in the context of mate choice have an open-ended preference for duty cycle that again drives males to produce extreme duty cycles. The potential effect of duty cycle on mate choice is consistent with the influence of signal power in male *A. grisella* on mate choice (Jang and Greenfield 1996). Thus, in *B. trigona* and *C. arizonensis*, natural selection and sexual selection may both select for high duty cycle signals but at different times and in separate contexts (defense and sex).

Many moth species use ultrasound for intraspecific communication (Conner 1999; Nakano et al. 2015). These include members of the Pyralidae, Erebidae, Geometridae, Nolidae, and Crambidae (Nakano et al. 2013, 2015; Greenfield 2014). The number of species discovered to use tymbal sounds in a courtship context continues to increase. In some tiger moth species, the courtship begins distantly with the emission of pheromones and the sound is produced during close-range interactions prior to copulation (Conner 1987; Sanderford et al. 1998). In contrast, *Syntomeida epilais* (Erebidae: Arctiinae) shows acoustic activity with both sexes producing signals at long-distance, in a kind of sexual dialog that promotes mating (Sanderford and Conner 1995). These examples indicate that acoustic courtship varies among lepidopterans and that it evolved much more frequently than previously thought.

In sonar jamming species like *B. trigona* the role of sound production has been well described in a predator–prey context (Corcoran et al. 2009, 2011; Corcoran and Conner 2012). This species produces ultrasound in response to bat cries at a duty cycle (43.8%) that is among the highest recorded for any tiger moth species (Barber and Conner 2006; Corcoran et al. 2010, 2011). As described above, a high duty cycle is critical for effective jamming (Miller 1991). Another closely related species that produces high duty cycle signals in its defensive repertoire is *C. arizonensis* (Dowdy and Conner 2019). Despite the growing interest in understanding the role of sound in intraspecific communication in lepidopterans (Nakano et al. 2009, 2013, 2015) the mating behavior of sonar-jamming species has not been described. Sullivan-Beckers and Cocroft (2010) devised an elegant method for determining the relative roles of female mate choice, male–male competition, and signal transmission on sexual selection in the treehopper, *Enchenopa binotata*, that communicates through substrate-borne vibration. As a first step, we have utilized several components of their methodology that are relevant to and tractable in our system, that is, the characterization of the variation in male signal duty cycle, the measurement of female preference for the duty cycle of male courtship signals, and the selection of male mates based on duty cycle “phenotype.” It is important to note that females of these species (unlike the treehoppers mentioned above) mate more than once (Y. Fernandez, personal communication) and direct male–male competition is minimal since female choice is expressed as the sequential assessment of individual males approaching a female. We address two hypotheses: the first is that females will show an open-ended preference for male courtship sounds with high duty cycles, and second that this preference will result in female mate choice for males with high duty cycle “phenotypes.”

To our knowledge this is the first study of intraspecific acoustic communication in tiger moths capable of jamming bat sonar, and it provides evidence supporting the parallel action of natural and sexual selection in at least one of these species.

## Materials and methods

### Research location and animals

Behavioral and playback experiments were conducted between July 17 and August 15 during 2017, 2018, and 2019 at the Southwestern Research Station (American Museum of Natural History), 7 km southwest of Portal, Arizona. The GPS coordinates of the field site are 31°53′00.30″ N 109°12′27.20″ W; elevation 1650 m. Adult individuals of *B. trigona* and *C. arizonensis* were collected from sheets illuminated with 15 W ultraviolet “quantum” lights (Leptraps.com; F15T8QBL) and mercury vapor lights set on the station grounds. Moths were separated by sex and held in cylindrical mesh containers (30 D × 31 H cm) for up to 24 h prior to experimentation. Animals were fed with 30% sucrose solution *ad libitum*. Sample sizes for the different experiments are indicated in Table 1.

**Table 1:** Total number of trials performed for each experiment and the number of individuals used per trial

	Breakdown by species							
	*Bertholdia trigona*				*Carales arizonensis*			
Experiment	Number of trials	Number of success	♀_s_/trial	♂_s_/trial	Number of trials	Number of success	♀_s_/trial	♂_s_/trial
Sound production in courtship	13	5/13	1	3-4	10	7/10	1	1
Variation in male duty cycle	30	N/A	N/A	1	12	N/A	N/A	1
Female preference for male duty cycle	54	N/A	1	N/A	N/A	N/A	N/A	N/A
Female mate choice	25	7/25	1	3^a^	8	8/8	1	2

*Notes*: The information is separated by species. For those experiments involving the observation of the courtship behavior, the number of successful trials is specified.

a In two of the female mate choice trials only two males were used instead of three, which decreased the number of tested males to 73 instead of 75 individuals. Neither of those two trials resulted in a successful mating.

### Sound production during mating

The courtship behavior of *B. trigona* and *C. arizonensis* was observed between the 19:00 and 6:00 h Pacific Standard Time (PST) and recorded to confirm the production of sound during courtship. For *B. trigona*, multiple individuals from both sexes (one female and three to four males) were placed in a mesh cage (28 W × 30 L × 25 H cm). This configuration was used to increase the likelihood of interactions between individuals. Mating behavior observations continued until a female accepted a male or rejected him by flying away. Once a successful pair formed, it was removed from the cage, and isolated in a separate mesh container constructed from a Styrofoam drinking cup and nylon fabric to continue monitoring for sound production during copulation. No individuals were used more than once. For *C. arizonensis*, it was unnecessary to promote physical interactions because pair formation occurred within minutes under laboratory conditions. Two individuals (one female and one male) were placed in a cylinder mesh cage (14 D × 17 H cm). In both species, the courtship was monitored using a video camera (model Sony 4K handycam HDR-HC9) in night vision mode. Acoustic emissions were continuously recorded with an ultrasonic microphone connected to an Ultrasound Gate 416H device (Avisoft Bioacoustics, Berlin, Germany), operated by a computer running Avisoft RECORDER USGH, sampling at 250 kHz. The microphone was located above the container, facing down toward the animals, at 20 cm from the container’s center. In the case of *B. trigona*, audio recordings started at the beginning of the experiment while multiple individuals were interacting and continued after a successful pair formed.

### Variation in male duty cycle

The variation in male duty cycle was analyzed for both species, *B. trigona* and *C. arizonensis*, by recording their modulation cycles (MCs) in response to tactile stimulation. Tactile stimulation consisted of gently touching the abdominal segments with a brush. Audio recordings were performed using the same experimental setup described above and males were held by the wings folded above the thorax using a hemostat. This procedure has been successfully used before to record sounds from tiger moths and did not cause any discernible change in their acoustic responses (Dowdy and Conner 2019). The ultrasonic microphone was placed perpendicular to the midline of the moth body at 10 cm from the thorax (where sound-producing organs are located), facing the right side. Every male was tested only once because intraindividual variation in duty cycle among a variety of tiger moth species has been found to be low in previous studies (Dowdy and Conner 2019), and so we do not examine it here. The duty cycle produced by each individual was determined by calculating the average click duration multiplied by the number of clicks produced in a sliding 100 ms time window. Reported duty cycles are derived from the time window containing the highest number of moth clicks from among all trials performed with a given individual. All audio recordings were analyzed in Avisoft SASLab Pro v5.2. Signals were identified in the audio recordings using an automatic detection method developed by Avisoft Bioacoustics and the measurements were performed in the oscillograms of the signals. Differences in the variance of male duty cycle between *B. trigona* and *C. arizonensis* individuals were tested with a *Z*-test.

### Female preference for male duty cycle: acoustic stimulation and audio recordings

Ultrasound playback files were generated in Matlab R2015a (The MathWorks, Inc., Natick, MA, USA). Each stimulus was derived from a natural MC previously recorded from *B. trigona* (at a 250 kHz sample rate; N. J. Dowdy, unpublished data) and altered using a custom Matlab script. This script was written to create simulated MCs with duty cycles of 10%, 25%, and 45% (Fig. 1C) (calculation assumes 100 ms window, 0.3 ms click duration, and a 4 ms interval between successive MCs), while maintaining other key acoustic characteristics defined in previous work such as sweep frequency, inter-cycle silent interval, and the active and passive half-MC durations (Corcoran et al. 2010). These values were chosen to cover the range of “low,” “medium,” and “high” duty cycle known to be produced by tiger moths (Barber and Conner 2006; Corcoran et al. 2010). The final stimuli consisted of five categories, including MCs of 10%, 25%, and 45% duty cycle, while silence and a white noise stimulus were used as negative and positive controls. Stimuli were composed of 600 ms acoustic emissions separated by 2.4 s of silent period. Each stimulus category was presented at least twice to *B. trigona* females.

Stimulation files were played back in random order with an AT 100 ultrasonic speaker (Binary Acoustic Technology), located 20 cm in front of the moth in the same horizontal plane. Ultrasonic clicks were broadcast with a peak equivalent sound pressure level of 80 dB, as measured with a quarter inch Microtech Geffel microphone (model MK 301) at 20 cm, connected to a conditioning measuring amplifier (Microtech Geffel MN-921). This amplitude is similar to that described for *B. trigona*’s sounds during an anti-bat response (Corcoran et al. 2010).

Female acoustic responses (production of their typical MCs) to artificial duty cycle were recorded during playback experiments using the same experimental setup described above. We determined the female preference for each duty cycle stimulus, as the number of presentations that elicited a response from the total number of presentations. In this case, the ultrasonic microphone was also placed at 10 cm from the thorax, perpendicular to the midline of the moth body. During acoustic recordings, moths were restrained by the wings with a hemostat. Moths were stimulated to produce sound by playing signals at different duty cycles.

A generalized linear mixed model using the *glm* function from the *stats* package was utilized to assess differences in female acoustic response among stimuli with different duty cycles. The “female response” variable was treated as a binary variable with “1” indicating sound production and “0” indicating female silence. When females responded, they did so within at most 0.5 s of the presentation of male signals. Therefore, females were classified as “unresponsive” when no response was detected within the 2.4 s silent period between stimuli. The duty cycle variable was treated as a ranked variable with five categories: silence (indicating a 0% duty cycle signal); 10%, 25%, and 45% duty cycle clicks (indicating males with diverse acoustic capabilities); and white noise (which represented a positive control stimulus with 100% duty cycle). Tukey’s test for multiple comparisons was used for *post hoc* analysis.

The latency of female acoustic responses to the five duty cycle categories described above was also recorded to determine whether females responded more quickly to higher duty cycle signals. Latency was calculated as the time interval between the beginning of playback and the first emitted click by the female. We found that females frequently did not respond to the “silence” group, so these were omitted from our calculations. We also observed that females often fail to respond to lower duty cycle signals and so these trials cannot be assigned a latency value. This is problematic for a direct statistical comparison of latencies between groups, as the high numbers of unresponsive females in the lower duty cycle groups would not be accounted for in such an analysis. To address this, we analyzed these results using a Cox proportional hazards regression model with the *survival* and *survminer* packages in R (Jahn-Eimermacher et al. 2011; Kassambara et al. 2019; Therneau 2020). This method examines the hazard rate (i.e., female response rate), proportional to a reference group. We chose the 45% duty cycle group as the reference level, as this is similar to the natural duty cycles produced by male moths. Therefore, the proportional hazards rate (i.e., “female response rate ratio”) for this group is defined as 1 and the results for all other groups are expressed as a ratio of this value (e.g., a mean proportional hazard rate of 2 indicates that females exposed to that duty cycle had a two-fold higher likelihood to produce a response over a given time period, relative to the 45% duty cycle group). Groups containing females which produce responses more quickly (i.e., with shorter latencies) than the reference group generate greater proportional hazards rates (i.e., “female response rate ratio”) and those that respond more slowly (i.e., with longer latencies) generate lower proportional hazards rates.

### Female mate choice experiments

To verify the role of sound production in this sexual context, we examined the effect of male duty cycle on the mating success. Males were randomly assigned to one of three treatments and used in female mate choice experiments. The treatments were divided into three “phenotypes”: (1) males able to produce normal duty cycle signals (S++), (2) males with reduced duty cycle (S+), and (3) males unable to produce sound (S−). Male moths from all treatments were placed in individual vials and chilled for 5 min in an ice bath before the surgery. The S++ group was removed from the ice and no further manipulations were performed. A sham control group was not included in this study, based on previous experiments (Dowdy and Conner 2016) showing that sham operations had no effect on sound production. The S+ and S− conditions were accomplished by puncturing the tymbals on the male metathorax with an entomological pin. For individuals in S+ condition, only half of the striated band (sound producing portion of the tymbal) on each side was affected, effectively reducing duty cycle, while for the S− group both tymbals were completely removed. The space underneath the moth’s tymbal organ is occupied by an air-filled chamber, so this ablation procedure did not cause any discernible injury or loss of hemolymph. All surgical operations were performed under a stereomicroscope 1 day before the behavioral tests. Males acoustic “phenotype” was confirmed after manipulation with an ultrasonic microphone.

Each *B. trigona* female was confined with three males (one per condition), except for two trials where only two males were tested (see Table 1). Mating activity was visually monitored to score outcomes. For each interaction, two possible outcomes were considered, (1) female rejected all the tested males or (2) female successfully mated with one male. Whenever scenario (2) took place, animals were isolated after the initiation of copulation. Once copulation was completed, female preference was determined by visual inspection of male tymbals under the microscope. From this examination, we were able to determine the male’s corresponding treatment (“S++,” “S+,” and “S−”). Female’s preference rate was calculated in relation to these three treatments.

A 2 × 3 Freeman–Halton extension of the Fisher’s exact probability test was used to test for differences in the frequency of female’s mate acceptance among males with different acoustic abilities (Freeman and Halton 1951). The predictor variable, male condition, was categorical with three levels (S++, S+, and S−). Data from seven trials (including 7 females and 21 males), where females successfully mated with one of the presented males, was included in this analysis (see Supplementary Table S1). Multiple pairwise comparisons were performed as a *post hoc* analysis, to test for differences in female’s mate acceptance between male conditions (S++, S+, and S−).

Similar mate choice experiments were performed in *C. arizonensis*. In this case, because the accessibility to individual males was low, males were only grouped in two conditions S++ and S−. Female’s mate choice was measured based on those two treatments. Fisher’s exact test was utilized to compare the frequency of female’s mate acceptance among clicking and muted males.

### Statistical analyses: general

Statistical analyses of observation data were performed in R version 3.5.2. Means are reported with the standard deviation of the mean. Where *P*-values were adjusted, we opted for the conservative Bonferroni correction method when performing multiple comparisons. Adjusted *P*-values greater than 1 are reported as 1. The standard alpha of 0.05 was used.

## Results

We here, document sound production in the courtship of *B. trigona* and *C. arizonensis*. We measure the variation in the max duty cycle produced between males. We measure female preference to male signals that vary in duty cycle. Last, we measure female mate choice for males that differ in high duty cycle “phenotype.”

### Sound production during courtship

In nature, courtship of these two moth species is likely initiated through the release of a female sex pheromone as evidenced by the presence of well-developed tubular pheromone glands in *B. trigona* (Beccacece 2015) and pulsatile pheromone releasing behavior in *C. arizonensis* (W. E. Conner, personal communication). There is no indication of male courtship pheromones or the elaborate scent disseminating structures (coremata) common in other arctiine species (Beccacece 2015). The normal behavioral sequence for courtship is truncated in our cages since following a female pheromone plume is not possible. Nevertheless, we found that *B. trigona* (both females and males) produce ultrasonic broadband clicks during close-range courtship behavior (Fig. 1B). Sound production is usually initiated by males and females join in. From a total of 13 females, 38% mated successfully (5/13). Tymbal sounds were detected in all interactions. When the female accepted a male, sound sometimes continued during copulation. The average duration of copulation was 5.3 h (min. 3.8 h and max. 6.0 h) during which a spermatophore is transferred from the male to female. Like other tiger moth species, sexual activity in *B. trigona* occurs at night (from sunset to 5:00 am PST). Only one mating was initiated at sunrise (5:43 am PST) and extended until late morning.

For *C. arizonensis* experiments, 7 of 10 tested females mated. In this species, pair formation (i.e., time elapsed since the animals were placed in the container until copulation begins) occurred in less than an hour (between 3 and 50 min). Acoustic communication was detected in 70% of the mating attempts and sound was produced only immediately prior to copulation. The courtship display in this species was also restricted to nocturnal hours and the copulation always lasted more than 6 h.

### Variation in male duty cycle

**Fig. 2:**
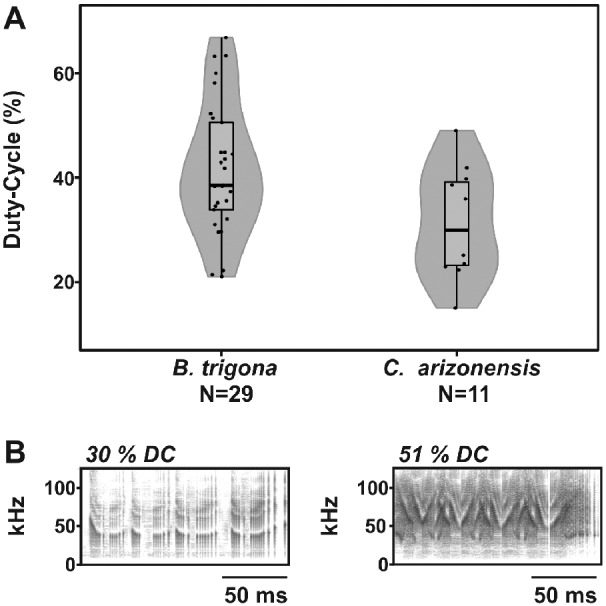
Variability of the male signals produced in response to tactile stimulation. (**A**) Violin plot of the duty cycles recorded from *B. trigona* and *C. arizonensis* males. Actual data from each tested individual are displayed as points jittered along the midline of their respective box plot. The gray curve represents the kernel distribution of the data. Box plot upper and lower hinges represent the 25th and 75th percentiles of their respective distributions. The 50th percentile (median) is shown as a thicker black line between the hinges. Tukey-style whiskers extend from each hinge to the most extreme value within 1.5 * IQR (inter-quartile range). (**B**) Spectrograms of the ultrasonic clicks produced by two individuals of *B. trigona* at different duty cycles. These emissions are examples of the observed variability and their duty cycles are close to the IQR limits of the species.

Male MCs were recorded in response to tactile stimulation from *B. trigona* (*N* = 30) and *C. arizonensis* (*N* = 12). The average observed for *B. trigona* was 41 ± 13% with a high of 67%. *Carales arizonensis* had a mean of 30 ± 11% and a maximum of 49% (Fig. 1A). We examined the intraspecific variation of emitted duty cycle for both species (Fig. 2A). Exemplar spectrograms demonstrating this variability within *B. trigona* are shown in Fig. 2B. *Bertholdia trigona* individuals showed a significantly greater range of duty cycles in their repertoire than *C. arizonensis* (*Z*-test: *P*-value = 0.01, *z* = 2.48, 95% CI = [2.3, 19.6]).

### Female preference for male duty cycle

**Fig. 3:**
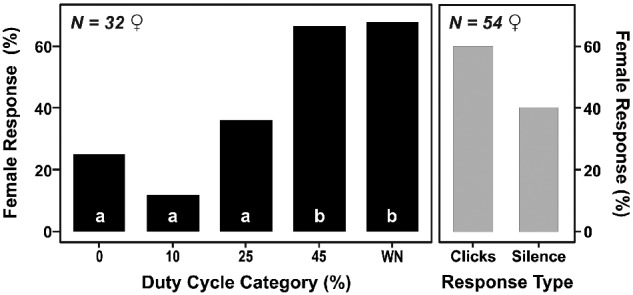
Effect of duty cycle on the acoustic response of *B. trigona* females. The right panel represents the percentage of individuals from the 54 tested females that produced clicks in response to acoustic stimulation. The left panel shows the response from clicking females (*N* = 32) to different duty cycles. Females were more acoustically active to higher duty cycles (Tukey’s test, *P* < 0.005).

To measure female preference for male signal duty cycle we presented five artificially generated signals with different duty cycles and measured female acoustic responses during playback experiments. We examined the audio recordings from 54 females and found that 60% of individuals (*n* = 32/54) produced sound in response to acoustic stimulation (Fig. 3). Females responded significantly more often to 45% duty cycle and white noise (100% duty cycle) (Tukey’s test, *P* < 0.001). Female clicks were detected in 67% of the presentations of 45% duty cycle signals and in 68% of white noise stimulations. Females responded less often to silence, low (10%), and moderate (25%) duty cycle stimuli. The response of females to white noise could be expected since tymbal sound and white noise share broadband frequency characteristics and since the internal temporal structure of tymbal sound is not resolvable by the moth ear (Sanderford et al. 1998).

**Fig. 4:**
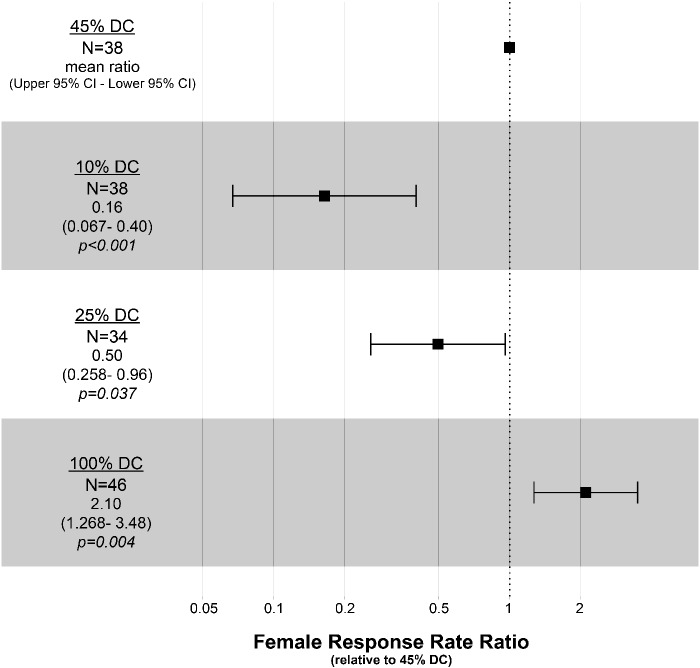
Duty cycle effect on female response rate within *B. trigona*. The rates of female acoustic responses to each simulated duty cycle are given, relative to the 45% duty cycle group. Mean response rates are given as squares with error bars indicating their 95% confidence intervals. Sample sizes, as well as values for mean rates, upper and lower confidence intervals, and *P*-values are given under each group. Relative to the 45% duty cycle group, which is most similar to the natural duty cycles of male *B. trigona*, the most extreme duty cycle (100%) had the highest response rate, whereas lower duty cycle (i.e., 10% and 25%) had significantly lower response rates.

Higher duty cycles also reduced female response latency, with females exhibiting a 50% higher response rate to 45% versus 25% duty cycles (95% CI: 0.260, 0.960; *P* = 0.03). The 10% duty cycle group’s rate of response was only 16% that of the 45% duty cycle group (95% CI: 0.067, 0.400; *P* < 0.001). However, the most extreme duty cycle elicited the most rapid female responses. The 100% duty cycle stimulus (white noise) had a 2.1-fold increased rate of female response relative to the 45% duty cycle stimulus (95% CI: 1.268, 3.482; *P* < 0.01) (Fig. 4).

The female responses to male duty cycle were distinctly different from those seen when playing back bats echolocation signals of equal intensity (Y. Fernandez, personal communication). Responses to bat cries are frequently accompanied by evasive maneuvers (head tilts and leg extension). None of these were seen when we stimulated with conspecific male sounds. Responses to bats also tend to continue throughout and after playback; this was not seen in female responses to male signals. In general, females’ responses to conspecifics had a lower number of MCs.

### Female mate choice for different duty cycle “phenotypes”

**Fig. 5:**
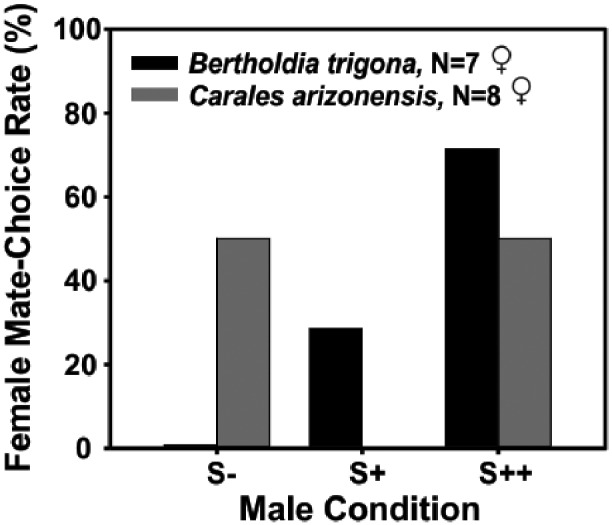
Females’ mate choice among males able to produce different duty cycle signals. Results are shown for individuals of *B. trigona* (females: *N* = 7; males: *N* = 21) for each male acoustic condition: S++ (high duty cycle clicks), S+ (moderate duty cycle clicks), and S− (no clicks). There were no *C. arizonensis* (females: *N* = 8; males: *N* = 16) assigned to the S+ group. Only the interactions that resulted in successful mating are included.

Female choice experiments demonstrated that acoustic communication is critical for mating success in *B. trigona*. We observed 25 females interacting with 73 males distributed across three experimental treatments (S−, S+, and S++). Seven of the observed attempts (28%) resulted in a mating. Most of these females, 71%, selected males producing high duty cycle (S++ group), whereas only 29% accepted a S+ male (Freeman–Halton extension Fisher’s exact test for male treatment: *P* = 0.003). Muted males (S− group) were never selected as a mate (Fig. 5). According to our results females chose S++ males more often than S+ males (Fisher’s exact test: *P* = 0.029) and muted males (Fisher’s exact test: *P* = 0.004).

For *C. arizonensis*, females accepted any approaching male regardless of whether they produced sound. We observed the interactions between 8 females and 16 males distributed in two acoustic treatment groups (S− and S++). Females accepted clicking and muted males each, in 50% of the cases (Fig. 5), suggesting that males were not selected based on their acoustic emissions in this species.

## Discussion

Arctiine moths are known to use sound in two contexts. The first is in a defensive context, where it can be used to warn of distastefulness and/or jam the sonar bat. Bats impose strong selective pressure on tiger moth’s duty cycle, driving its evolution toward extreme values. This idea has been supported by previous studies showing that species clicking at low duty cycle (e.g., *Pygarctia roseicapitis* and *Cisthene martini*, from Dowdy and Conner 2016) have a higher capture rate than *B. trigona*, which produce high duty cycle signals (Corcoran et al. 2009, 2010, 2011). Although we knew that natural selection molds the temporal design of moth clicks, we here explored arctiine sounds in the context of sexual selection. Our two hypotheses were supported. Female *B. trigona* displays an open-ended preference for male courtship sounds with high duty cycles. This preference ultimately resulted in female mate choice of males with high duty cycle “phenotypes.”

It is well known that across a wide range of species, sexual selection generates patterns in male acoustic signals through female choice (Greenfield 2002; Gerhardt and Huber 2002). In this study, *B. trigona* male signals showed variability in their duty cycle, suggesting that this acoustic trait could be a target for sexual selection. This substantial variation exhibited in male duty cycle (21–67%, see Fig. 2) might be considered the “signal range” of this character (e.g., Gerhardt and Huber 2002; Amezquita et al. 2011). We also determined the corresponding “recognition range” (i.e., the duty cycle range that elicited a female response; Gerhardt and Huber 2002) in our playback experiments. Females showed a large “recognition range” (from 10% to 100% duty cycle, see Fig. 3) with a strong preference toward higher duty cycle signals (45% and 100%). Females display a larger “recognition range” than the males “signal range,” and they prefer exaggerated signals (e.g., the 100% white noise stimuli used) over those actually produced by males (Greenfield 2016). According to Greenfield (2016), this difference between “signal range” and “recognition range” implies that female choice imposes selection on males’ sounds, shifting their duty cycle toward higher values. In *B. trigona*, natural and sexual selection work in parallel to drive the evolution of extreme duty cycles. Due to the small sample sizes in these experiments, however, our conclusion must be tentative.

An alternative view of our data is to interpret the female preference curve as evidence for male–male competition, whereby males with higher duty cycles can “freeze” the female and prevent her from leaving (Nakano et al. 2010a, 2010b). While certainly possible we do not find this mechanism compelling because in the natural context females (with few exceptions) are silently releasing a sex pheromone and it is the concentration of the female pheromone that actually triggers male sound production downwind of the female (Rodgers 1991). At this point the female is unlikely to flee/depart but instead frequently displays a receptive posture in response to male sound production (wing-raising). The switch from anti-bat behavior to courtship behavior is likely a function of releasing and sensing the female sex pheromone (Skals et al. 2005) in arctiines.

The role of sound in the courtship of *C. arizonensis* is less clear. In this species, acoustic emissions were limited to the final moments prior to copulation and during some successful interactions, no sound was detected. Their mean duty cycle was significantly lower and the variation around the mean is also lower than in *B. trigona* (see Fig. 2). Female mate choice experiments with *C. arizonensis* indicate that acoustic communication is not essential for mating. It is possible that chemical and acoustic signals in this species are redundant as in *Cycnia tenera* (Conner 1987) or that the role of acoustic signals has been lost as in *Pyrrharctia isabella* (Krasnoff and Yager 1988).

Recent phylogenetic analyses report that *B. trigona* and *C. arizonensis* are members of two separate clades traditionally classified within the subtribe Phaegopterina (Zaspel et al. 2014; Zenker et al. 2017). Members of these two clades were not previously known to produce sound during courtship. Our findings have important implications for our understanding of the evolutionary history of sexual communication in tiger moths. Acoustic courtship has either evolved multiple times independently within the subfamily or it originated earlier than previously thought. A broad survey of acoustic sexual behavior, in combination with a robust, densely-sampled phylogeny, is needed to place our results in an historical framework.

## Supplementary Material

obaa046_Supplementary_Data

## References

[obaa046-B1] Amezquita A , Flechas SV, Lima AP, Gasser H, Hodl W. 2011 Acoustic interference and recognition space within a complex assemblage of dendrobatid frogs. Proc Natl Acad Sci U S A 108:17058–63. 21969562 10.1073/pnas.1104773108 PMC3193210

[obaa046-B2] Andersson M. 1994 Sexual selection. Princeton (NJ): Princeton University Press. p. 620.

[obaa046-B3] Barber JR , Conner WE. 2006 Tiger moth responses to a simulated bat attack: timing and duty cycle. J Exp Biol 209:2637–50. 16809455 10.1242/jeb.02295

[obaa046-B4] Beccacece HM. 2015 Filogenia del Grupo Halysidota (Lepidoptera, Erebidae, Arctiinae) en el Neotrópico, con la revisión taxonómica de las especies argentinas [PhD dissertation]. Universidad Nacional de Córdoba. 276 pp.

[obaa046-B5] Conner WE. 1987 Ultrasound: its role in the courtship of the arctiid moth, *Cycnia tenera*. Experientia 43:1029–31.

[obaa046-B6] Conner WE. 1999 Un chant d’appel amoureux’: acoustic communication in moths. J Exp Biol 202:1711–23. 10359675 10.1242/jeb.202.13.1711

[obaa046-B7] Corcoran AJ , Barber JR, Conner WE. 2009 Tiger moth jams bat sonar. Science 325:325–7. 19608920 10.1126/science.1174096

[obaa046-B8] Corcoran AJ , Conner WE, Barber JR. 2010 Anti-bat tiger moth sounds: form and function. Curr Zool 56:358–69.

[obaa046-B9] Corcoran AJ , Barber JR, Hristov NI, Conner WE. 2011 How do tiger moths jam bat sonar? J Exp Biol 214:2416–25. 21697434 10.1242/jeb.054783

[obaa046-B10] Corcoran AJ , Conner WE. 2012 Sonar jamming in the field: effectiveness and behavior of a unique prey defense. J Exp Biol 215:4278–87. 23175526 10.1242/jeb.076943

[obaa046-B11] Dowdy NJ , Conner WE. 2016 Acoustic aposematism and evasive action in select chemically defended arctiine (Lepidoptera: Erebidae) species: nonchalant or not? PLoS ONE 11:e0152981. 27096408 10.1371/journal.pone.0152981 PMC4838332

[obaa046-B12] Dowdy NJ , Conner WE. 2019 Nonchalant flight in tiger moths (Erebidae: Arctiinae) is correlated with unpalatability. Front Ecol Evol 7.

[obaa046-B13] Emlen DJ. 2014 Animal weapons: the evolution of battle. Henry Holt and Company p. 288.

[obaa046-B14] Freeman GH , Halton JH. 1951 Note on exact treatment of contingency, goodness of fit and other problems of significance. Biometrika 38:141–9. 14848119

[obaa046-B15] Gerhardt HC , Huber F. 2002 Acoustic communication in insects and anurans: common problems and diverse solutions. University of Chicago Press p. 531.

[obaa046-B16] Greenfield MD. 2002 Signalers and Receivers: mechanisms and evolution of arthropod communication. Oxford University Press p. 427.

[obaa046-B17] Greenfield MD. 2014 Acoustic communication in the nocturnal Lepidoptera In: Hedwig B, editor. Insect hearing and acoustic communication. Berlin: Springer p. 81–100.

[obaa046-B18] Greenfield MD. 2016 Sexual selection In: Jeremy AD, Ring CT, editors. Pheromone communication in moths: evolution, behavior, and application. Oakland: University of California Press. p. 401.

[obaa046-B19] Jahn-Eimermacher A , Lasarzik I, Raber J. 2011 Statistical analysis of latency outcomes in behavioral experiments. Behav Brain Res 221:271–5. 21397635 10.1016/j.bbr.2011.03.007 PMC3103828

[obaa046-B20] Jang Y , Greenfield MD. 1996 Ultrasonic communication and sexual selection in wax moths: female choice based on energy and asynchrony of male signals. Anim Behav 51:1095–106.

[obaa046-B21] Kassambara A , Kosinski M, Biecek P. 2019 survminer: drawing survival curves using ‘ggplot2’. R package version 0.4.6. Available from: https://CRAN.R-project.org/package=survminer.

[obaa046-B22] Krasnoff SB , Yager DD. 1988 Acoustic response to a pheromonal cue in the arctiid moth *Pyrrharctia Isabella*. Physiol Entomol 13:433–40.

[obaa046-B23] Miller LA. 1991 Arctiid moth clicks can degrade the accuracy of range difference discrimination in echolocating big brown bats, *Eptesicus fuscus*. J Comp Physiol A 168:571–9. 1920158 10.1007/BF00215079

[obaa046-B24] Nakano R , Takanashi T, Fujii T, Skals N, Surlykke A, Ishikawa Y. 2009 Moths are not silent, but whisper ultrasonic courtship songs. J Exp Biol 212:4072–8. 19946086 10.1242/jeb.032466

[obaa046-B25] Nakano R , Takanashi T, Skals N, Surlykke A, Ishikawa Y. 2010a Ultrasonic courtship songs of male Asian corn borer moths assist copulation attempts by making the females motionless. Physiol Entomol 35:76–81.

[obaa046-B26] Nakano R , Takanashi T, Skals N, Surlykke A, Ishikawa Y. 2010b To females of a noctuid moth, male courtship songs are nothing more than bat echolocation calls. Biol Lett 6:582–4. 20219743 10.1098/rsbl.2010.0058 PMC2936132

[obaa046-B27] Nakano R , Takanashi T, Surlykke A, Skals N, Ishikawa Y. 2013 Evolution of deceptive and true courtship songs in moths. Sci Rep 3:2003. 23788180 10.1038/srep02003 PMC3687589

[obaa046-B28] Nakano R , Takanashi T, Surlykke A. 2015 Moth hearing and sound communication. J Comp Physiol A 201:111–21. 10.1007/s00359-014-0945-8 25261361

[obaa046-B29] Neelon DP , Rodrìguez RL, Höbel G. 2019 On the architecture of mate choice decisions: preference functions and choosiness are distinct traits. Proc R Soc B 286:20182830. 10.1098/rspb.2018.2830 PMC6408907 30963823

[obaa046-B30] Pryke SR , Andersson S. 2002 A generalized female bias for long tails in a short-tailed widowbird. Proc R Soc B 269:2141–6. 10.1098/rspb.2002.2131 PMC1691140 12396489

[obaa046-B31] Rodgers MR. 1991 Sex attraction in *Cycnia tenera* Hbn. (Lepidoptera: Arctiidae): chemical characterization of the pheromone blend and description of the pheromone gland [MS thesis]. Wake Forest University. 131 pp.

[obaa046-B32] Rodriguez RL , Greenfield MD. 2004 Behavioral context regulates dual function of hearing in ultrasonic moths: bat avoidance and pair formation. Physiol Entomol 29:159–68.

[obaa046-B33] Sanderford MV , Conner WE. 1995 Acoustic courtship communication in *Syntomeida epilais* Wlk. (Lepidoptera: Arctiidae, Ctenuchinae). J Insect Behav 8:19–31.

[obaa046-B34] Sanderford MV , Coro F, Conner WE. 1998 Courtship behavior in *Empyreuma affinis* roths. (Lepidoptera, Arctiidae, Ctenuchinae): acoustic signals and tympanic organ response. Naturwissenschaften 85:82–7.

[obaa046-B35] Skals N , Anderson P, Kanneworff M, Lofsted C, Surlykke A. 2005 Her odours make him deaf: crossmodel modulation and hearing in a male moth. J Exp Biol 208:595–601. 15695752 10.1242/jeb.01400

[obaa046-B36] Sullivan-Beckers L , Cocroft RB. 2010 The importance of female choice, male–male competition, and signal transmission as causes of selection on male mating signals. Evolution 64:3158–71. 20624180 10.1111/j.1558-5646.2010.01073.x

[obaa046-B37] Therneau T. 2020 A package for survival analysis in R. R package version 3.1-12. Available from: https://CRAN.R-project.org/package=survival.

[obaa046-B38] Tougaard J , Casseday JH, Covey E. 1998 Arctiid moths and bat echolocation: broad-band clicks interfere with neural responses to auditory stimuli in the nuclei of the lateral lemniscus of the big brown bat. J Comp Physiol A 182:203–15. 9463919 10.1007/s003590050171

[obaa046-B39] Zaspel JM , Weller SJ, Wardwell CT, Zahiri R, Wahlberg N. 2014 Phylogeny and evolution of pharmacophagy in tiger moths (Lepidoptera: Erebidae: Arctiinae). PLoS ONE 9:e101975. 25036028 10.1371/journal.pone.0101975 PMC4103773

[obaa046-B40] Zenker MM , Wahlberg N, Brehm G, Teston JA, Przybylowicz L, Pie MR, Freitas AVL. 2017 Systematics and origin of moths in the subfamily Arctiinae (Lepidoptera, Erebidae) in the neotropical region. Zool Scr 46:348–62.

