# Supplementary material for: Extreme Duty Cycles in the Acoustic Signals of Tiger Moths: Sexual and Natural Selection Operating in Parallel
Source: Integr Org Biol. 2021 Jan 5;2(1):obaa046. doi: 10.1093/iob/obaa046 (PMC7810578; doi:10.1093/iob/obaa046)
Supplement: obaa046_Supplementary_Data [file obaa046_supplementary_data.docx]

Table S1. A 2x3 contingency table for female mate choice experiment. Mate choice data from seven *Bertholdia trigona* females which were presented males possessing different acoustic conditions are displayed. Males were classified as muted (unable to produce sound), moderate clickers (with reduced duty cycles) and normal clickers (with both tymbals functionals able to produce high duty cycles). These acoustic conditions were experimentally obtained by tymbal ablation. Totals are given in bold.

|  |  | |  |  |
| --- | --- | --- | --- | --- |
|  | ***Female mate choice outcome*** | | | |
| **Male Condition** | **Rejected** | **Mated** | | **Total** |
| Muted | 7 | 0 | | **(7)** |
| Reduced Clicker (S+) | 6 | 1 | | **(7)** |
| Normal Clicker (S++) | 1 | 6 | | **(7)** |
| Total | **(14)** | **(7)** | | **[21]** |
